# Tracking Recombination Events That Occur in Conjugative Virulence Plasmid p15WZ-82_Vir during the Transmission Process

**DOI:** 10.1128/mSystems.00140-20

**Published:** 2020-07-14

**Authors:** Xuemei Yang, Lianwei Ye, Edward Wai-Chi Chan, Rong Zhang, Sheng Chen

**Affiliations:** a Department of Infectious Diseases and Public Health, Jockey Club College of Veterinary Medicine and Life Sciences, City University of Hong Kong, Kowloon, Hong Kong; b State Key Lab of Chemical Biology and Drug Discovery, Department of Applied Biology and Chemical Technology, The Hong Kong Polytechnic University, Hung Hom, Hong Kong; c Department of Clinical Laboratory, School of Medicine, Second Affiliated Hospital of Zhejiang University, Zhejiang, Hangzhou, China; Institute for Systems Biology

**Keywords:** *Klebsiella pneumoniae*, hypervirulence plasmid, conjugative, plasmid evolution, homologous recombination

## Abstract

We recently reported the recovery of a conjugative virulence plasmid, p15WZ-82_Vir, from a clinical Klebsiella variicola strain. In this study, we found that several new plasmid types were generated due to genetic rearrangement. Partial integration of plasmid p15WZ-82_Vir with existing plasmids such as resistance plasmids by different homologous recombination events was observable in three recipient strains. Such recombination events enable the formation of various types of mosaic plasmids simultaneously carrying virulence-encoding and antibiotic resistance-encoding genes as well as genes involved in plasmid conjugation, which promote transmission of various virulence-encoding and resistance-encoding elements among pathogens. Our data also suggest that these conjugative events may play an integral role in the development of novel mosaic plasmids, which is vital for plasmid evolution.

**IMPORTANCE** Although they are often nonconjugative, large virulence plasmids are increasingly detected in clinical K. pneumoniae and contribute to the hypervirulence phenotype of this organism. In this study, we demonstrated that the virulence-encoding region that originated from virulence plasmid pLVPK actively interacted with different types of plasmids via homologous recombination to generate new conjugative plasmids. This report provides insights into the evolution of self-transmissible plasmids carrying genetic elements encoding both hypervirulent and multidrug-resistant phenotypes, which facilitate the rapid development of clinical K. pneumoniae strains that are hypervirulent and multidrug resistant.

## Abstract (precis)

Although they are often nonconjugative, large virulence plasmids are increasingly detected in clinical K. pneumoniae and contribute to the hypervirulence phenotype of this organism. In this study, we demonstrated that the virulence-encoding region that originated from virulence plasmid pLVPK actively interacted with different types of plasmids via homologous recombination to generate new conjugative plasmids. This report provides insights into the evolution of self-transmissible plasmids carrying genetic elements encoding both hypervirulent and multidrug-resistant phenotypes, which facilitate the rapid development of clinical K. pneumoniae strains that are hypervirulent and multidrug resistant.

## INTRODUCTION

As an invasive pathogen, Klebsiella pneumoniae employs a variety of virulence factors to evade and inhibit the host immune response, colonize specific sites, and acquire nutrients (1). Along with the conserved chromosomal islands which encode virulence factors, large virulence plasmids are increasingly being found to play a role in determining the virulence level of K. pneumoniae. pKP100, a 180-kbp plasmid which encodes the aerobactin iron uptake system and the mucoid phenotype, was first identified and found to play an important role in regulating virulence expression in K. pneumoniae K1 and K2 isolates (2, 3). A 219-kbp virulence plasmid, pLVPK, was isolated from a highly virulent clinical K2 K. pneumoniae isolate (4). This plasmid carries several virulence-associated genes, including the mucoid phenotype regulatory gene *rmpA* and its homolog *rmpA2*, gene clusters *iucABCDiutA* and *iroBCDN* which encode the iron acquisition systems aerobactin and salmochelin siderophore, the *fepBC* genes which originated from Mesorhizobium loti and encode the ABC iron transporter, and the Escherichia coli *fecIRA* genes which encode the iron uptake system. A recent report showed that a 200-kbp virulence plasmid which harbors a number of virulence-encoding genes resembling those located in pLVPK but which is structurally not identical to pLVPK is commonly present in hypervirulent clinical K. pneumoniae isolates (4). The molecular mechanism underlying the conjugation of this kind of virulence plasmid remains poorly understood.

Bacteria evolve rapidly by acquiring exogenous genetic elements via horizontal gene transfer (HGT), a process which enables recipient bacterial strains to acquire beneficial genotypes from multiple organisms (5, 6). A successful HGT event includes not only the introduction of foreign DNA into the cytoplasm of a recipient cell but also replication and functional expression of the acquired genetic element as part of the recipient genome. Conjugative plasmids play an important role in HGT by encoding proteins that are involved in important functional activities in cell-cell interaction (7). Another advantage of genetic transfer through plasmid uptake is that it provides an autonomously replicating genetic element which could become established in the recipient strains (5). In a previous study, we reported the discovery of a virulence-encoding plasmid, p15WZ-82_Vir, which formed as a result of incorporation of a 100-kb fragment of plasmid pLVPK into a conjugative IncFIB plasmid (8). This plasmid was demonstrated to be conjugated from the parental Klebsiella variicola strain to E. coli strain EC600 and to *Klebsiella* strains of different multilocus sequence types (ST), promoting dissemination of virulence-encoding elements among Gram-negative bacterial pathogens (8). To track the details of the process of evolution of plasmid p15WZ-82_Vir during the process of transmission to K. pneumoniae strains of different ST, a total of 13 pairs of recipient strains and transconjugant strains were subjected to further genetic characterization. Taking advantage of third-generation sequencing technology, this study investigated the recombination events that occur in plasmid p15WZ-82_Vir during the process of conjugative transfer to different bacterial strains.

## RESULTS AND DISCUSSION

**FIG 1:**
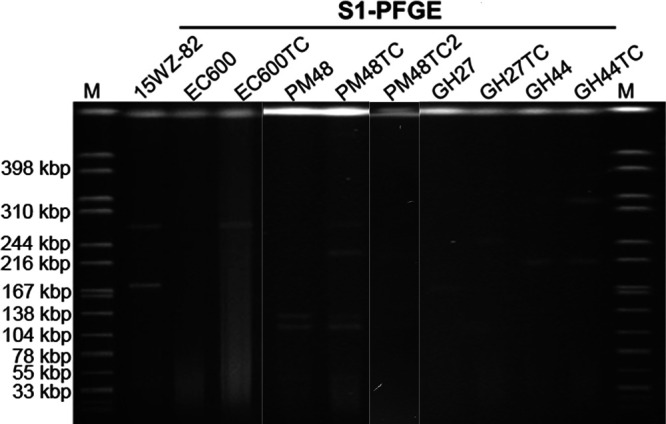
S1-PFGE analysis of parental strains and transconjugants. S1-PFGE analysis of strain 15WZ-82, recipient strain E. coli EC600, its corresponding transconjugant E. coli EC600TC, and the corresponding transconjugants obtained when EC600-TC was used as the donor was performed. PM48TC2 was generated after serial culturing of PM48TC. The S1-PFGE was repeated twice for all strains with the same results.

In a previous study, we reported the discovery of a virulence-encoding plasmid, p15WZ-82_Vir, which was demonstrated to be able to be conjugated from parental *K. variicola* strain 15WZ-82 to E. coli strain EC600 and different K. pneumoniae strains (Table 1) (8). To investigate the microevolution events that occur in plasmid p15WZ-82_Vir during the transmission process, 13 pairs of recipients and transconjugants were subjected to S1 nuclease pulsed-field gel electrophoresis (S1-PFGE) analysis. The results demonstrated that most of the transconjugants had acquired this 280-kbp plasmid (see Fig. S1 in the supplemental material). However, K. pneumoniae transconjugants of strains PM48, GH27, and GH44 exhibited unusual profiles (Fig. S1). The transconjugant of strain PM48 was found to have acquired another 230-kbp plasmid, in addition to the 280-kbp plasmid acquired from the donor strain. Strain GH27 was found to harbor a 170-kbp plasmid, and yet the size of the plasmid recovered from the corresponding transconjugant was 240 kbp. Likewise, the plasmid acquired by the transconjugant of strain GH44 was found to be as large as 320 kbp. After serial batch culturing for 2 weeks, the 280-kbp and 140-kbp plasmids disappeared and the newly generated 230-kbp plasmid remained in the transconjugant of stain PM48, PM48TC2; the 240-kbp and 320-kbp plasmids were found to be stable in the transconjugants of strain GH27 and strain GH44, respectively (Fig. 1). These observations indicated that a number of genetic rearrangement or recombination events might occur during transmission of plasmid p15WZ-82_Vir from the donor strain to the recipient strain and that the resulting plasmids could be stably inherited in the transconjugants. To decipher the nature of these events, all plasmids were extracted from the donor and recipient strains and subjected to whole-genome sequencing.

**TABLE 1:** Phenotypic and genotypic characteristics of K. pneumoniae transconjugants determined using EC600-TC1 as donor strain^*a*^

Strain ID	Bacterial species	Sequence type	MIC (μg/ml)										Presence of *rmpA2*	Conjugation efficiency
			CAZ	CTX	IPM	MEM	ETP	AK	CIP	PB	ATM	TE		
15WZ-82	*K. variicola*	ST595	4	8	8	16	16	4	<1	1	>128	>128	+	
EC600	E. coli		<1	<1	<1	<1	<1	4	<1	<0.5	<1	<1	−	
EC600-TC1	E. coli		<1	<1	<1	<1	<1	4	<1	<0.5	<1	>128	+	6.25E−08
14WZ-24	K. pneumoniae	ST11	>128	>128	>128	>128	>128	>128	>32	<0.5	>128	2	−	
14WZ-24TC	K. pneumoniae		>128	>128	>128	>128	>128	>128	>32	<0.5	>128	128	+	4.53E−04
HKU1	K. pneumoniae	ST716	>64	>64	<1	<1	<1	<2	64	2	>128	<2	−	
HKU1TC	K. pneumoniae		>64	64	<1	<1	<1	<2	64	2	>128	32	+	3.84E−04
HKU6	K. pneumoniae	ST101	>64	>64	<1	<1	<1	<2	8	1	>128	4	−	
HKU6TC	K. pneumoniae		>64	>64	<1	<1	<1	<2	16	1	>128	64	+	1.57E−02
HKU33	K. pneumoniae	ST307	>64	<2	<1	<1	<1	4	64	2	>128	<1	−	
HKU33TC	K. pneumoniae		>64	<2	<1	<1	<1	2	64	2	>128	64	+	1.95E−04
HKU41	K. pneumoniae	ST1326	>64	8	<1	<1	<1	>64	32	2	>128	<2	−	
HKU41TC	K. pneumoniae		>64	8	<1	<1	<1	>64	32	2	>128	32	+	1.77E−02
HKU45	K. pneumoniae	ST313	16	<2	<1	<1	<1	<2	16	2	>128	<2	−	
HKU45TC	K. pneumoniae		32	<2	<1	<1	<1	<2	8	2	>128	32	+	2.57E−04
HKU50	K. pneumoniae	ST16	>64	16	<1	<1	<1	4	>64	2	>128	4	−	
HKU50TC	K. pneumoniae		>64	16	<1	<1	<1	2	64	2	>128	128	+	1.78E−04
GH27	K. pneumoniae	ST37	4	8	<1	<1	<1	>64	>64	1	>128	<2	−	
GH27TC	K. pneumoniae		4	8	<1	<1	<1	>64	>64	1	>128	64	+	1.65E−04
GH44	K. pneumoniae	ST395	>64	16	<1	<1	<1	<2	>64	4	>128	<2	−	
GH44TC	K. pneumoniae		>64	16	<1	<1	<1	<2	>64	4	>128	64	+	8.40E−06
PM1	K. pneumoniae	ST15	<1	<1	<1	<1	<1	<2	>64	4	>128	<2	−	
PM1TC	K. pneumoniae		<1	<1	<1	<1	<1	<2	>64	4	>128	32	+	7.73E−05
PM7	K. pneumoniae	ST15	<1	<1	<1	<1	<1	<2	>64	2	>128	4	−	
PM7TC	K. pneumoniae		<1	<1	<1	<1	<1	<2	>64	2	>128	64	+	1.56E−06
PM28	K. pneumoniae	ST14	<1	<1	<1	<1	<1	<2	16	1	>128	<2	−	
PM28TC	K. pneumoniae		<1	<1	<1	<1	<1	<2	16	1	>128	32	+	9.37E−04
PM48	K. pneumoniae	ST35	>64	8	<1	<1	<1	<2	16	2	>128	<2	−	
PM48TC	K. pneumoniae		>64	16	<1	<1	<1	<2	16	2	>128	32	+	4.64E−06

a CAZ, ceftazidime; CTX, cefotaxime; ID, identifier; IPM, imipenem; MEM, meropenem; ETP, ertapenem; AK, amikacin; CIP, ciprofloxacin; PB, polymyxinB(E); ATM, aztreonam; TE, tellurite.

10.1128/mSystems.00140-20.1 FIG S1 S1-PFGE analysis of parental strains and transconjugants. S1-PFGE analyses of strain 15WZ-82, recipient strain E. coli EC600, its corresponding transconjugant E. coli EC600TC, and the corresponding transconjugants obtained when EC600TC was used as donor were repeated twice for all strains with the same results. The arrow indicates the expected size of p15WZ-82 at 280 kbp, and the asterisks (*) indicate the usual profiles of transconjugants of strains GH27, GH44, and PM48. Download FIG S1, TIF file, 1.2 MB. Copyright © 2020 Yang et al. 2020 Yang et al. This content is distributed under the terms of the Creative Commons Attribution 4.0 International license.

**FIG 2:**
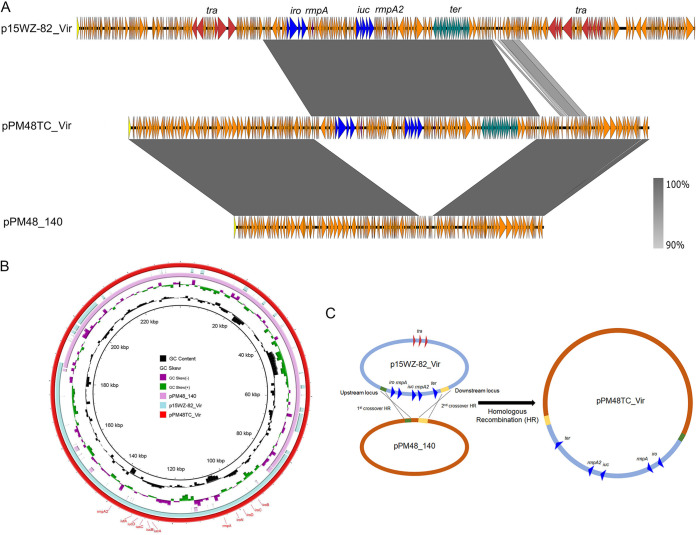
Evolution of p15WZ-82_Vir to pPM48TC_Vir during the transmission process. (A) Alignment of plasmid pPM48TC_Vir with plasmid p15WZ-82_Vir and pPM48_140 by easyfig. (B) Alignment of plasmid pPM48TC_Vir with plasmid p15WZ-82_Vir and pPM48_140 by BRIG. (C) Illustration of the process of evolution of p15WZ-82_Vir to pPM48TC_Vir.

Strain PM48 was found to harbor two plasmids with sizes of 140,128 bp and 125,936 bp, respectively, which was consistent with the S1-PFGE result (Table 2). The 125,936-bp plasmid was an IncFII_K_ plasmid harboring several resistance genes and designated pPM48_125 (Fig. S2A). Plasmid pPM48_125 exhibited highest similarity to plasmid pCTXM15_020019 (GenBank accession no. CP028553.2) from K. variicola strain WCHKP19, with coverage of 92% and identity of 100%. Resistance genes *aac(6’)-Ib-cr*, *arr3*, *dfrA27*, *aadA16*, and *sul1* were found to be located in an integron. This plasmid also harbors *mph*(A), *bla*_TEM-1_, and *bla*_CTX-M-3_. Notably, plasmid pPM48_125 was found to contain *tra* genes which encode plasmid conjugation function. Further conjugation experimentation showed that this plasmid was able to conjugate to E. coli strain EC600. This kind of plasmid is easy to acquire and spreads resistance genes easily, representing a threat to human health. The 140,128-bp plasmid was an IncFIB_K_ plasmid and was designated pPM48_140 (Fig. 2; see also Fig. S2B). Plasmid pPM48_140 was found to contain no resistance genes and to show highest similarity to Klebsiella aerogenes strain NCTC9667 plasmid 2 (GenBank accession no. LR134207.1). However, the initially isolated PM48TC transconjugant strain was found to harbor four plasmids with sizes of 282,290 bp, 235,461 bp, 140,128 bp, and 125,936 bp, respectively. The coexistence of these four plasmids was demonstrated in both the S1-PFGE profiles and sequencing results. The sequences of the 140,128-bp and 125,936-bp plasmids in this transconjugant were found to be identical to those of the plasmids harbored by the parental PM48 strain without any mutation. The additional 282,290-bp plasmid was found to be identical to plasmid p15WZ-82_Vir. The 235,461-bp plasmid was found to be the result of a fusion between plasmids p15WZ-82_Vir and pPM48_140 (Fig. 2). Further sequence analysis showed that an 889-bp upstream homologous region and a 1,246-bp downstream homologous region were present in the two plasmids. Identification of these two homologous regions, which were located in two different plasmids, could help explain the evolution process of p15WZ-82. Briefly, the 103-kbp virulence-encoding fragment of p15WZ-82 was integrated into pPM48_140 and the other structural regions were excised from the two plasmids, resulting in formation of a 235,461-bp integrated virulence plasmid, pPM48TC_Vir (Fig. 2C). In addition to the large rearrangement, a small (<1%) ratio of single-nucleotide polymorphisms (SNPs) was also found to have occurred in this mosaic plasmid compared to parental plasmids. Interestingly, coexistence of the four plasmids was observed only in initial transconjugant PM48TC. After serial culturing, two parental plasmids were lost and only the mosaic plasmid remained in transconjugant PM48TC2. These findings indicated that the mosaic plasmid was more stable than the corresponding parental plasmids.

**TABLE 2:** Plasmid information corresponding to donor strain 15WZ-82, the three recipient strains, and the corresponding transconjugants

Strain ID	Plasmid ID	Plasmid type	Size (bp)	Resistance gene(s)	Presence of *tra* genes	GenBank no.	Similar plasmid(s) (GenBank no./% coverage/% identity)
15WZ-82	p15WZ-82_Vir	IncFIB	282,290	None	+	CP032355	K. pneumoniae strain KSB1_10J unnamed2 (CP024517/68/99)
	p15WZ-82_KPC	IncX5	41,874	*bla*_KPC-2_, *bla*_TEM-1_	**−**	CP032356	p13190-KPC (MF344555/97/99)
	p15WZ-82_res	IncFIB_K_/ IncFII	185,478	Class D beta-lactamase gene	+	CP032357	

PM48	pPM48_140	IncFIB_K_	140,128	None	**−**	MN543579	Klebsiella aerogenes strain NCTC9667 plasmid 2 (LR134207)
	pPM48_125	IncFII_K_	125,936	*aac(6’)-Ib-cr*, *arr3*, *dfrA27*, *aadA16*, *sul1*, *mph*(A), *bla*_TEM-1_, *bla*_CTX-M-3_	+	MN543580	pCTXM15_020019 (CP028553/92/100)

PM48TC	pPM48TC_Vir	IncFIB_K_	235,461	None	**−**	MN543581	Mosaic plasmid of p15WZ-82_Vir and pPM48_140
	pPM48TC_125	IncFII_K_	125,936	*aac(6’)-Ib-cr*, *arr3*, *dfrA27*, *aadA16*, *sul1*, *mph*(A), *bla*_TEM-1_, *bla*_CTX-M-3_	+	MN543583	Identical to pPM48_125

GH27	pGH27_175	IncFIB_K_/ IncHI1B	175,909	*aadA2*, *cmlA*, *aadA1*, *sul3*, *mef*(B), *sul1*, *aadA16*, *dfrA27*, *arr-3*, *aac(6’)Ib-cr*, *floR*	**−**	MN543571	pKPN-065 (CP015026/88/99) pF10AN_1 (CP026154/84/99)
	pGH27_70	IncFIA/ IncR	70,382	*bla*_DHA-1_, *qnrB4*, *qnrB6*, *sul1*, *sul2*, *strA*, *strB*	**−**	MN543572	pLA-64 (CP035381/76/100) p234 (CP021163/76/100) pR50-74 (CP040362/75/99)

GH27TC	pGH27TC_Vir	IncFIB_K_/ IncHI1B	250,183	*aadA2*, *cmlA*, *aadA1*, *sul3*, *mef*(B), *sul1*, *aadA16*, *dfrA27*, *arr-3*, *aac(6’)Ib-cr*, *floR*	**−**	MN543585	Mosaic plasmid of p15WZ-82_Vir and pGH27_175
	pGH27TC_70	IncFIA/ IncR	70,382	*bla*_DHA-1_, *qnrB4*, *qnrB6*, *sul1*, *sul2*, *strA*, *strB*	**−**	MN543584	Identical to pGH27_70

GH44	pGH44_43	IncR	43,389	*aadA2*, *cmlA*, *aadA1*, *sul3*, *mef*(B)	**−**	MN543574	K. pneumoniae strain 2e (CP040177/64/99), pEco-36682cz (MG557999/67/99)
	pGH44_216	IncFIB_K_/ IncFII_K_	216,159	*aph(3’)-Ia*, *mph*(A), *sul1*, *bla*_DHA-1_, *qnrB4*, *aadA16*, *dfrA27*, *arr3*, *tet*(D), *floR*, *bla*_CTX-M-27_	+	MN543573	K. pneumoniae strain FDAARGOS_444 plasmid unnamed1 (CP023943/88/100)

GH44TC	pGH44TC_Vir	IncFIB/ IncR	327,581	*aadA2*, *cmlA*, *aadA1*, *sul3*, *mef*(B)	+	MN543576	Mosaic plasmid of p15WZ-82_Vir and pGH44_43
	pGH44TC_216	IncFIB_K_/ IncFII_K_	216,159	*aph(3’)-Ia*, *mph*(A), *sul1*, *bla*_DHA-1_, *qnrB4*, *aadA16*, *dfrA27*, *arr3*, *tet*(D), *floR*, *bla*_CTX-M-27_	+	MN543577	Identical to pGH44_216

10.1128/mSystems.00140-20.2 FIG S2 Description of plasmids harbored by K. pneumoniae strains PM48, GH27, and GH44. (A) Alignment of plasmid pPM48_125 with plasmid pCTXM15_020019 (GenBank accession no. CP028553.2). (B) Alignment of plasmid pPM48_140 with Klebsiella aerogenes strain NCTC9667 plasmid 2 (GenBank accession no. LR134207.1). (C) Alignment of plasmid pGH27_175 with plasmid pKPN-065 (GenBank accession no. CP015026.1) and pF10AN_1 (GenBank accession no. CP026154.1). (D) Alignment of plasmid pGH27_70 with plasmid pLA-64 (GenBank accession no. CP035381.1), p234 (GenBank accession no. CP021163.1), and pR50-74 (GenBank accession no. CP040362.1). (E) Alignment of plasmid pGH44_43 with K. pneumoniae strain 2e plasmid unnamed (GenBank accession no. CP040177.1) and plasmid pEco-36682cz (GenBank accession no. MG557999.1). (F) Alignment of plasmid pGH44_216 with K. pneumoniae strain FDAARGOS_444 plasmid unnamed1 (GenBank accession no. CP023943.1). Download FIG S2, TIF file, 1.6 MB. Copyright © 2020 Yang et al. 2020 Yang et al. This content is distributed under the terms of the Creative Commons Attribution 4.0 International license.

**FIG 3:**
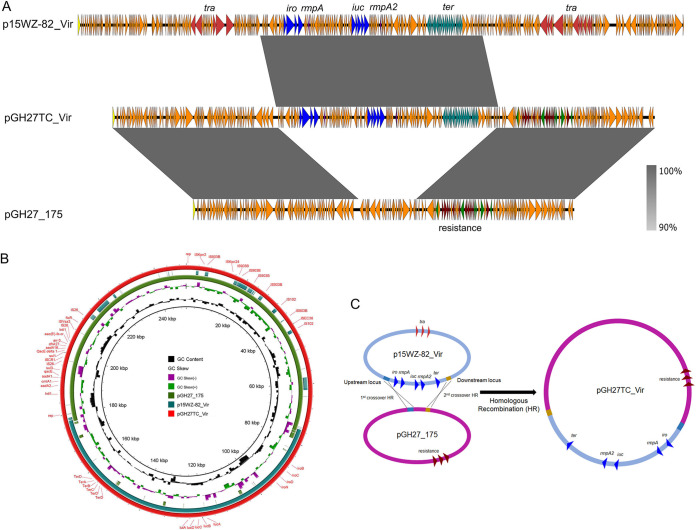
Evolution of p15WZ-82_Vir to pGH27TC_Vir during the transmission process. (A) Alignment of plasmid pGH27TC_Vir with plasmid p15WZ-82_Vir and pGH27_175 by easyfig. (B) Alignment of plasmid pGH27TC_Vir with plasmid p15WZ-82_Vir and pGH27_175 by BRIG. (C) Illustration of the process of evolution of p15WZ-82_Vir to pGH27TC_Vir.

Strain GH27 was found to harbor two plasmids with sizes of 175,909 bp and 70,382 bp, respectively, which was consistent with the S1-PFGE result (Table 2). The 175,909-bp plasmid was an IncFIB_K_/IncHI1B plasmid harboring several resistance genes and was designated pGH27_175 (Fig. 3; see also Fig. S2C). Plasmid pGH27_175 exhibited highest similarity to plasmid pKPN-065 (GenBank accession no. CP015026.1) and plasmid pF10AN_1 (GenBank accession no. CP026154.1), with identity to both of 99% and coverage of 88% and 84%, respectively. Both plasmids were recovered from K. pneumoniae strains and differed from pGH27_175 at the region harboring resistance genes. The resistance genes identifiable in this plasmid included *aadA2*, *cmlA*, *aadA1*, *sul3*, *mef*(B), *sul1*, *aadA16*, *dfrA27*, *arr-3*, *aac(6’)Ib-cr*, and *floR* and were found to be located in two integrons. The 70,382-bp plasmid was also a resistance-encoding plasmid that belonged to the IncFIA/IncR type and was designated pGH27_70 (Fig. S2D). Results of BLAST searches against the NCBI database showed that plasmid pGH27_70 exhibited 100% identity to and 76% coverage of plasmid pLA-64 (GenBank accession no. CP035381.1) from a Leclercia adecarboxylata strain and plasmid p234 (GenBank accession no. CP021163.1) from an Enterobacter hormaechei strain. The most similar plasmid from K. pneumoniae was plasmid pR50-74 (GenBank accession no. CP040362.1), with 75% coverage and 99% identity. The three plasmids shared similar backbones with plasmid pGH27_70 but differed with respect to the resistance genes. The resistance genes identifiable in this plasmid included *bla*_DHA-1_, *qnrB4*, *qnrB6*, *sul1*, *sul2*, *strA*, and *strB*, which were flanked by diverse insertion sequences. However, the corresponding transconjugant GH27TC strain was found to harbor two plasmids with sizes of 250,183 bp and 70,382 bp, respectively (Table 2). The 70,382-bp plasmid was identical to the one that the donor harbored without any mutation, whereas the 250,183-bp plasmid was found to be the result of a fusion between plasmids p15WZ-82_Vir and pGH27_175 and was designated pGH27TC_Vir (Fig. 3). Further sequence analysis showed that both plasmids harbored a 724-bp upstream homologous region and a 484-bp downstream homologous region, facilitating homologous recombination of the two plasmids. As a result, the 102-kbp virulence-encoding fragment of p15WZ-82_Vir was integrated into pGH27_175, whereas the other structural regions were excised from these two plasmids, resulting in formation of a 250,183-bp integrated virulence and multidrug resistance (MDR) plasmid (Fig. 3C). About 200 SNPs were also found in this mosaic plasmid.

**FIG 4:**
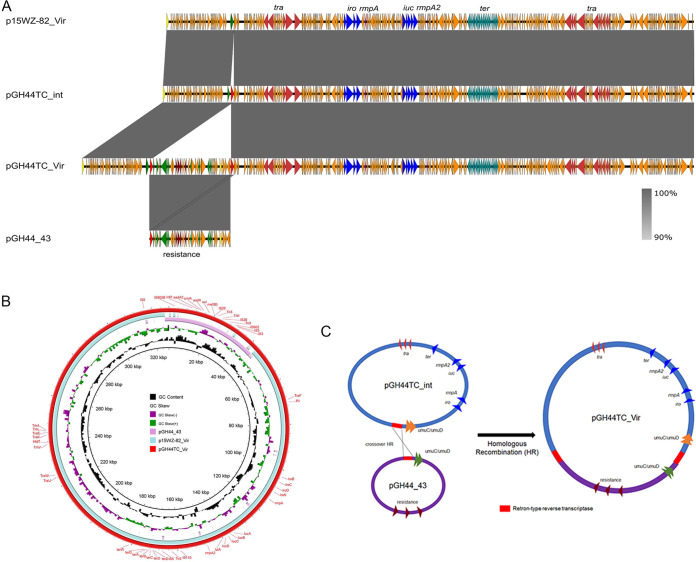
Evolution of p15WZ-82_Vir to pGH44TC_Vir during the transmission process. (A) Alignment of plasmid pGH44TC_Vir with plasmid p15WZ-82_Vir and pGH44_43 by easyfig. (B) Alignment of plasmid pGH44TC_Vir with plasmid p15WZ-82_Vir and pGH44_43 by BRIG. (C) Illustration of the process of evolution of p15WZ-82_Vir to pGH44TC_Vir.

Strain GH44 was found to harbor two plasmids with sizes of 216,159 bp and 43,389 bp, respectively (Table 2). The 43,389-bp plasmid was an IncR plasmid harboring the resistance genes *aadA2*, *cmlA*, *aadA1*, *sul3*, and *mef*(B) and was designated pGH44_43 (Fig. 4; see also Fig. S2E). Plasmid pGH44_43 exhibited highest similarity to a plasmid (GenBank accession no. CP040177.1) from K. pneumoniae strain 2e and plasmid pEco-36682cz (GenBank accession no. MG557999.1) from E. coli strain Eco-36682cz, with identity to both of 99% and coverage of 64% and 67%, respectively. All three plasmids were found to share similar plasmid backbones but differing mosaic resistance regions. The 216,159-bp plasmid was also a resistance plasmid belonging to the IncFIB_K_/IncFII_K_ type and was designated pGH44_216 (Fig. S2F). Plasmid pGH44_216 showed highest similarity to K. pneumoniae strain FDAARGOS_444 plasmid unnamed1 (GenBank accession no. CP023943.1), with identity of 100% and coverage of 88%. Plasmid pGH44_216 was found to harbor several resistance genes, including *aph(3′)-Ia*, *mph*(A), *sul1*, *bla*_DHA-1_, *qnrB4*, *aadA16*, *dfrA27*, *arr3*, *tet*(D), *floR*, and *bla*_CTX-M-27_, as well as heavy metal resistance gene clusters. Plasmid conjugation *tra* genes have been identified in plasmid pGH44_216, indicating that this plasmid might be conjugative. However, strain GH44TC was found to harbor four plasmids with sizes of 327,581 bp, 284,388 bp, 216,159 bp, and 43,389 bp, respectively, according to the sequencing result. The 216,159-bp and 43,389-bp plasmids were found to be identical to plasmids harbored by the recipient strain GH44 without any mutation. The 327,581-bp plasmid was found to be the result of a fusion between plasmids p15WZ-82_Vir and pGH44_43, and five SNPs have been identified in this mosaic plasmid (Fig. 4). The 284,388-bp plasmid was found to contain an extra 2,098-bp fragment encoding a Retron-type reverse transcriptase compared to virulence plasmid p15WZ-82_Vir. Further sequence analysis showed that the upstream region of the joint fragment was an IS*200*/IS*605* family element transposase and that the downstream region was an error-prone repair protein, UmuC/UmuD. Interestingly, a duplicated Retron-type reverse transcriptase coding area was identified in the fused virulence plasmid, which could help explain the evolution process of p15WZ-82_Vir (Fig. 4C). A region which encoded the Retron-type reverse transcriptase in pGH44_43 was inserted into p15WZ-82_Vir, resulting in pGH44TC_int. A crossover homologous recombination event then took place in this homologous region, leading to integration of these two plasmids and formation of a 327,581-bp integrated virulence-encoding and resistance-encoding plasmid, pGH44TC_Vir. However, the coexistence of these four plasmids was found to be a temporary phenomenon after we incubated strain GH44TC for a longer time. The 284,388-bp and 43,389-bp plasmids disappeared and the 327,581-bp and 216,159-bp plasmids remained in strain GH44TC. Only the 320-kbp and 210-kbp plasmids were observed by S1-PFGE in strain GH44TC after serial culturing, suggesting that previously identified 284,388-bp plasmid pGH44TC_int might represent an intermediate product of the integration process and might be lost after serial culturing. Even though the benefits associated with generation of this mosaic plasmid were unknown, there is no doubt that this plasmid could promote transmission of virulence-encoding and resistance-encoding elements simultaneously, posing a great threat to human health.

Alignment of the pGH27TC_Vir and pPM48TC_Vir mosaic plasmids showed that the homologous regions involved in the partial integration event were found in both plasmids, although a low level of structural difference was observed (Fig. S3). The actual homologous region in p15WZ-82_Vir is unknown because the independent plasmids involved in the fusion process in strain 15WZ-82 had disappeared during evolution. The mechanism that we had proposed in our previous study also involved homologous recombination with an upstream homologous tellurite resistance locus and a downstream homologous region. We found that the downstream homologous region identified in p15WZ-82_Vir was highly similar to that identified in plasmids pGH27TC_Vir and pPM48TC_Vir. Another homologous region in pGH27TC_Vir and pPM48TC_Vir was found to be located upstream of the tellurite resistance locus, which was identified as a homologous region in p15WZ-82_Vir. Through this homologous recombination mechanism, the 100-kbp virulence-encoding region from plasmid pLVPK was integrated with a conjugative plasmid, resulting in formation of the integrated plasmid p15WZ-82_Vir. The virulence-encoding region from plasmid p15WZ-82_Vir was also found to be integrated into different plasmids which harbored the homologous regions. Furthermore, only the integrated plasmids were identified in strain 15WZ-82 and GH27TC, indicating that the original plasmids were lost during the evolution process. The observation of the disappearance of original plasmids in strain PM48TC after serial culturing represented directly obtained evidence. Together, these results indicated that this 100-kbp virulence-encoding region that had originated from virulence plasmid pLVPK could actively interact with different types of plasmids via homologous recombination, generating mosaic plasmids of various sizes. Such recombination events therefore generated mosaic plasmids that were self-transmissible and that carried virulence-encoding and multidrug resistance-encoding genes, which could promote cotransmission of virulence-encoding and resistance-encoding elements. Mosaic plasmids are highly abundant, and nearly half of all plasmids have been found to be mosaic (9). Mosaic plasmids are associated with spread of antibiotic resistance genes. Our study depicted the details of the process of the generation of mosaic plasmids, which broadens our understanding of plasmid evolution. Most importantly, the generation of different conjugative plasmids containing the virulence region of the virulence plasmid might dramatically increase transmission efficiency and the host spectrum and might enable the rapid transmission of hypervirulence-encoding plasmids among Gram-negative bacteria.

10.1128/mSystems.00140-20.3 FIG S3 Alignment of plasmids pLVPK, pGH27TC_Vir, and pPM48TC_Vir using plasmid p15WZ-82_Vir as a reference. The homologous regions identified in pGH27TC_Vir and pPM48TC_Vir are indicated. Download FIG S3, TIF file, 0.6 MB. Copyright © 2020 Yang et al. 2020 Yang et al. This content is distributed under the terms of the Creative Commons Attribution 4.0 International license.

### Conclusion.

In conclusion, the recombination events that occurred in plasmid p15WZ-82_Vir during the transmission process were characterized. Partial plasmid integration were observed when recipient strains had acquired plasmid p15WZ-82_Vir. Taking three independent strains as examples, mechanisms underlying plasmid integration were illustrated. Our data will broaden the understanding of the mechanisms by which virulence plasmids evolve. The generation and spread of mosaic plasmids which carry both virulence and antibiotic resistance pose great threats to human health.

## MATERIALS AND METHODS

### Bacterial strains.

Information regarding the sources of the strains studied in this work can be found in our previous study (8). K. variicola strain 15WZ-82 was recovered from a sputum sample from a 65-year-old female patient in the general ward of a hospital in Zhejiang province, China, in 2015. All K. pneumoniae strains used in this study were recovered from clinical samples of patients admitted to different hospitals located in mainland China and the Hong Kong SAR.

### Conjugation assay.

Conjugation of the virulence plasmid of strain 15WZ-82 was performed using rifampin-resistant E. coli strain EC600 as the recipient. Briefly, both strains were cultured to the logarithmic phase (optical density [OD] of ∼0.6) at 37°C in Luria-Bertani (LB) broth. Then 100 μl of culture of the donor cells and 400 μl of culture of the recipient cells were mixed and inoculated carefully onto a 0.45-μm-pore-size membrane placed on the surface of a LB agar plate. After incubation at 37°C overnight, bacteria on the membrane were collected, resuspended in saline solution, and serially diluted. The diluted culture was spread on China Blue agar plates containing 2 μg/ml potassium tellurite (K_2_TeO_3_) and 600 μg/ml rifampin for selection of transconjugants. The presence of *rmpA2* as a marker gene of virulence plasmids in transconjugants was determined by PCR as previously described (10). The MIC profiles of the transconjugants were also determined for differentiation between the transconjugants and the donor strains. S1 nuclease pulsed-field gel electrophoresis (S1-PFGE) was performed as described previously (11) to confirm acquisition of this plasmid by the recipient strain. Conjugation was then performed by using E. coli transconjugant EC600-TC1 as the donor and ciprofloxacin-resistant K. pneumoniae strains as the recipients. MacConkey agar plates containing 2 or 8 μg/ml potassium tellurite (K_2_TeO_3_) and 2 μg/ml ciprofloxacin were used to select transconjugants depending on the tellurite MIC of the recipient K. pneumoniae strains. The genetic identity and phenotypic features of the transconjugants were also confirmed as described above. To calculate the conjugation efficiency, the diluted culture obtained after conjugation was also spread on plates containing only 600 μg/ml rifampin for strain EC600 as the recipient and only 2 μg/ml ciprofloxacin for *Klebsiella* strains as the recipients to determine the total number of recipient cells. The conjugation efficiency was calculated as the number of transconjugants divided by the number of recipient cells.

### Stability of acquired plasmids in transconjugants.

The transconjugants were purified by streaking of the single colony on fresh LB agar plates for three repeats. Serial culturing of the pure transconjugants were carried out for 2 weeks in LB broth by transferring 100 μl of bacterial culture to 10 ml of fresh LB (100-fold dilution) every 12 h. The stability of acquired plasmids in transconjugants was determined by plating bacterial cultures on LB plates containing 8 μg/ml potassium tellurite (K_2_TeO_3_). S1-PFGE analysis of the selected colonies was also performed to confirm the presence of the plasmids in transconjugants after serial culturing.

### Whole-genome sequencing and assembly.

Plasmid DNA was extracted and sequenced via the use of a 150-bp paired-end Illumina NextSeq 500 platform (Illumina, San Diego, CA) and a long-read Oxford Nanopore Technologies MinION platform (Nanopore, Oxford, United Kingdom) as previously described (8). Both short and long reads were subjected to *de novo* hybrid assembly using Unicycler v0.4.7 (12). Assembled genome sequences were annotated with RAST v2.0 (13) and Prokka v1.12 (14).

### Bioinformatics analysis.

Virulence genes were identified by searches against the BIGSdb Klebsiella genome database (http://bigsdb.pasteur.fr/klebsiella/klebsiella.html). The BLAST command lines, with an 80% coverage cutoff, were used to map genome sequences against antibiotic resistance genes and plasmid replicons, the databases of which were obtained from the Center for Genomic Epidemiology (http://www.genomicepidemiology.org/). Alignments of plasmids with similar structures were generated by the use of BLAST Ring Image Generator (BRIG) version 0.95.22 (15) and Easyfig_win_2.1 (16).

### Data availability.

Complete sequences of the plasmids of strains GH27, GH44, and PM48 and their corresponding transconjugants have been deposited in the GenBank database under accession numbers MN543571 to MN543585. All other data related to this study are available upon request.

## References

[1] Li B, Zhao Y, Liu C, Chen Z, Zhou D 2014 Molecular pathogenesis of *Klebsiella pneumoniae*. Future Microbiol 9:1071–1081. doi:10.2217/fmb.14.48. 25340836

[2] Nassif X, Sansonetti PJ 1986 Correlation of the virulence of *Klebsiella pneumoniae* K1 and K2 with the presence of a plasmid encoding aerobactin. Infect Immun 54:603–608. doi:10.1128/IAI.54.3.603-608.1986. 2946641 PMC260211

[3] Nassif X, Fournier JM, Arondel J, Sansonetti PJ 1989 Mucoid phenotype of *Klebsiella pneumoniae* is a plasmid-encoded virulence factor. Infect Immun 57:546–552. doi:10.1128/IAI.57.2.546-552.1989. 2643575 PMC313131

[4] Lev AI, Astashkin EI, Kislichkina AA, Solovieva EV, Kombarova TI, Korobova OV, Ershova ON, Alexandrova IA, Malikov VE, Bogun AG, Borzilov AI, Volozhantsev NV, Svetoch EA, Fursova NK 2018 Comparative analysis of *Klebsiella pneumoniae* strains isolated in 2012–2016 that differ by antibiotic resistance genes and virulence genes profiles. Pathog Glob Health 112:142–151. doi:10.1080/20477724.2018.1460949. 29708041 PMC6056825

[5] Thomas CM, Nielsen KM 2005 Mechanisms of, and barriers to, horizontal gene transfer between bacteria. Nat Rev Microbiol 3:711–721. doi:10.1038/nrmicro1234. 16138099

[6] Pinilla-Redondo R, Cyriaque V, Jacquiod S, Sørensen SJ, Riber L 2018 Monitoring plasmid-mediated horizontal gene transfer in microbiomes: recent advances and future perspectives. Plasmid 99:56–67. doi:10.1016/j.plasmid.2018.08.002. 30086339

[7] Stokes HW, Gillings MR 2011 Gene flow, mobile genetic elements and the recruitment of antibiotic resistance genes into Gram-negative pathogens. FEMS Microbiol Rev 35:790–819. doi:10.1111/j.1574-6976.2011.00273.x. 21517914

[8] Yang X, Chan EW, Zhang R, Chen S 2019 A conjugative plasmid that augments virulence in *Klebsiella pneumoniae*. Nat Microbiol 4:2039–2043. doi:10.1038/s41564-019-0566-7. 31570866

[9] Pesesky MW, Tilley R, Beck D 2019 Mosaic plasmids are abundant and unevenly distributed across prokaryotic taxa. Plasmid 102:10–18. doi:10.1016/j.plasmid.2019.02.003. 30797764

[10] Gu D, Dong N, Zheng Z, Lin D, Huang M, Wang L, Chan EW, Shu L, Yu J, Zhang R, Chen S 2018 A fatal outbreak of ST11 carbapenem-resistant hypervirulent *Klebsiella pneumoniae* in a Chinese hospital: a molecular epidemiological study. Lancet Infect Dis 18:37–46. doi:10.1016/S1473-3099(17)30489-9. 28864030

[11] Huang Y, Yu X, Xie M, Wang X, Liao K, Xue W, Chan EW, Zhang R, Chen S 2016 Widespread dissemination of carbapenem-resistant *Escherichia coli* sequence type 167 strains harboring blaNDM-5 in clinical settings in China. Antimicrob Agents Chemother 60:4364–4368. doi:10.1128/AAC.00859-16. 27114282 PMC4914679

[12] Wick RR, Judd LM, Gorrie CL, Holt KE 2017 Unicycler: resolving bacterial genome assemblies from short and long sequencing reads. PLoS Comput Biol 13:e1005595. doi:10.1371/journal.pcbi.1005595. 28594827 PMC5481147

[13] Brettin T, Davis JJ, Disz T, Edwards RA, Gerdes S, Olsen GJ, Olson R, Overbeek R, Parrello B, Pusch GD, Shukla M, Thomason JA III, Stevens R, Vonstein V, Wattam AR, Xia F 2015 RASTtk: a modular and extensible implementation of the RAST algorithm for building custom annotation pipelines and annotating batches of genomes. Sci Rep 5:8365. doi:10.1038/srep08365. 25666585 PMC4322359

[14] Seemann T 2014 Prokka: rapid prokaryotic genome annotation. Bioinformatics 30:2068–2069. doi:10.1093/bioinformatics/btu153. 24642063

[15] Alikhan NF, Petty NK, Ben Zakour NL, Beatson SA 2011 BLAST Ring Image Generator (BRIG): simple prokaryote genome comparisons. BMC Genomics 12:402. doi:10.1186/1471-2164-12-402. 21824423 PMC3163573

[16] Sullivan MJ, Petty NK, Beatson SA 2011 Easyfig: a genome comparison visualizer. Bioinformatics 27:1009–1010. doi:10.1093/bioinformatics/btr039. 21278367 PMC3065679

