# Supplementary figures and images for: Tracking Recombination Events That Occur in Conjugative Virulence Plasmid p15WZ-82_Vir during the Transmission Process
Source: mSystems. 2020 Jul 14;5(4):e00140-20. doi: 10.1128/mSystems.00140-20 (PMC7363002; doi:10.1128/mSystems.00140-20)

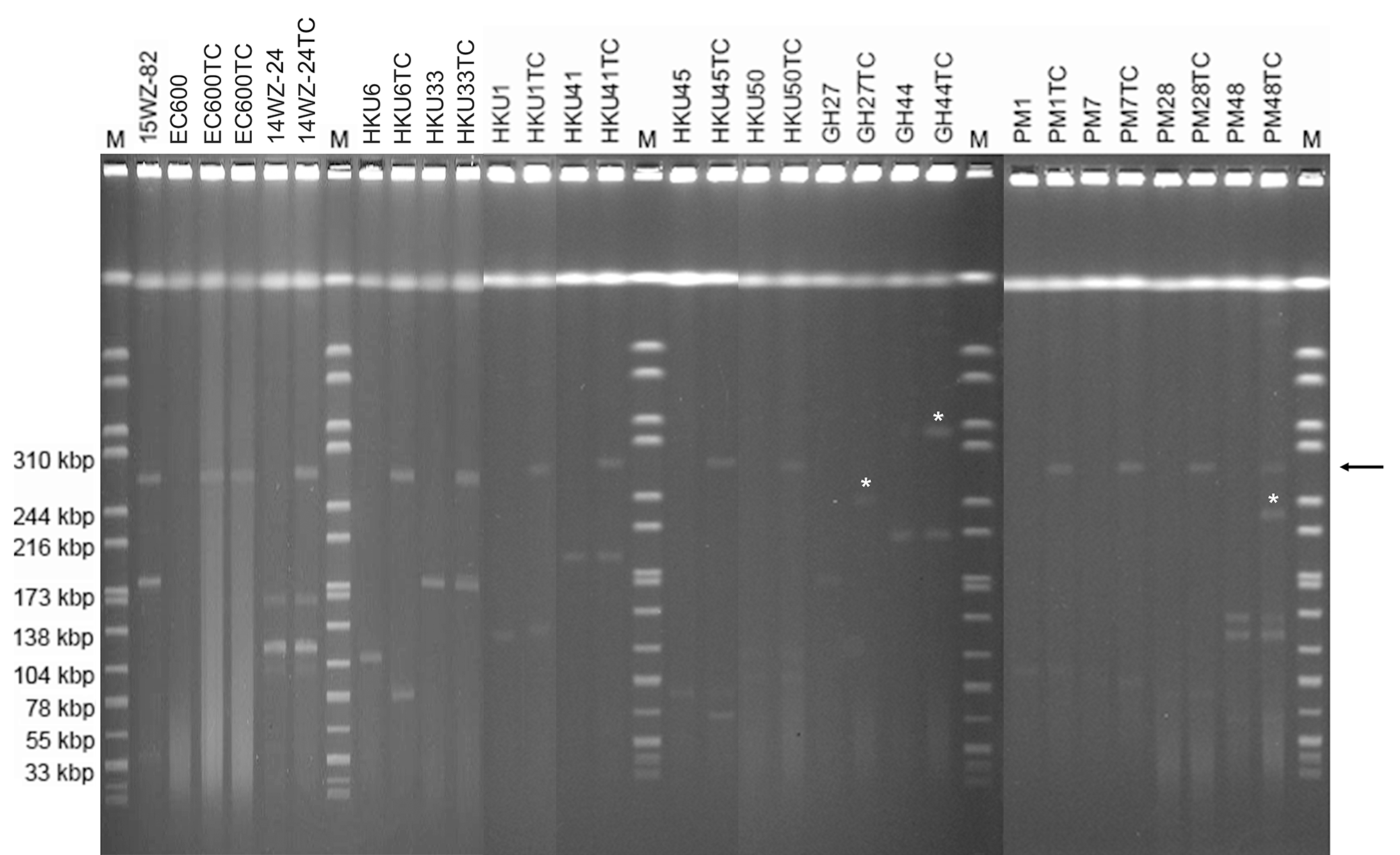

Supplement: FIG S1 [file mSystems.00140-20-sf001.tif]

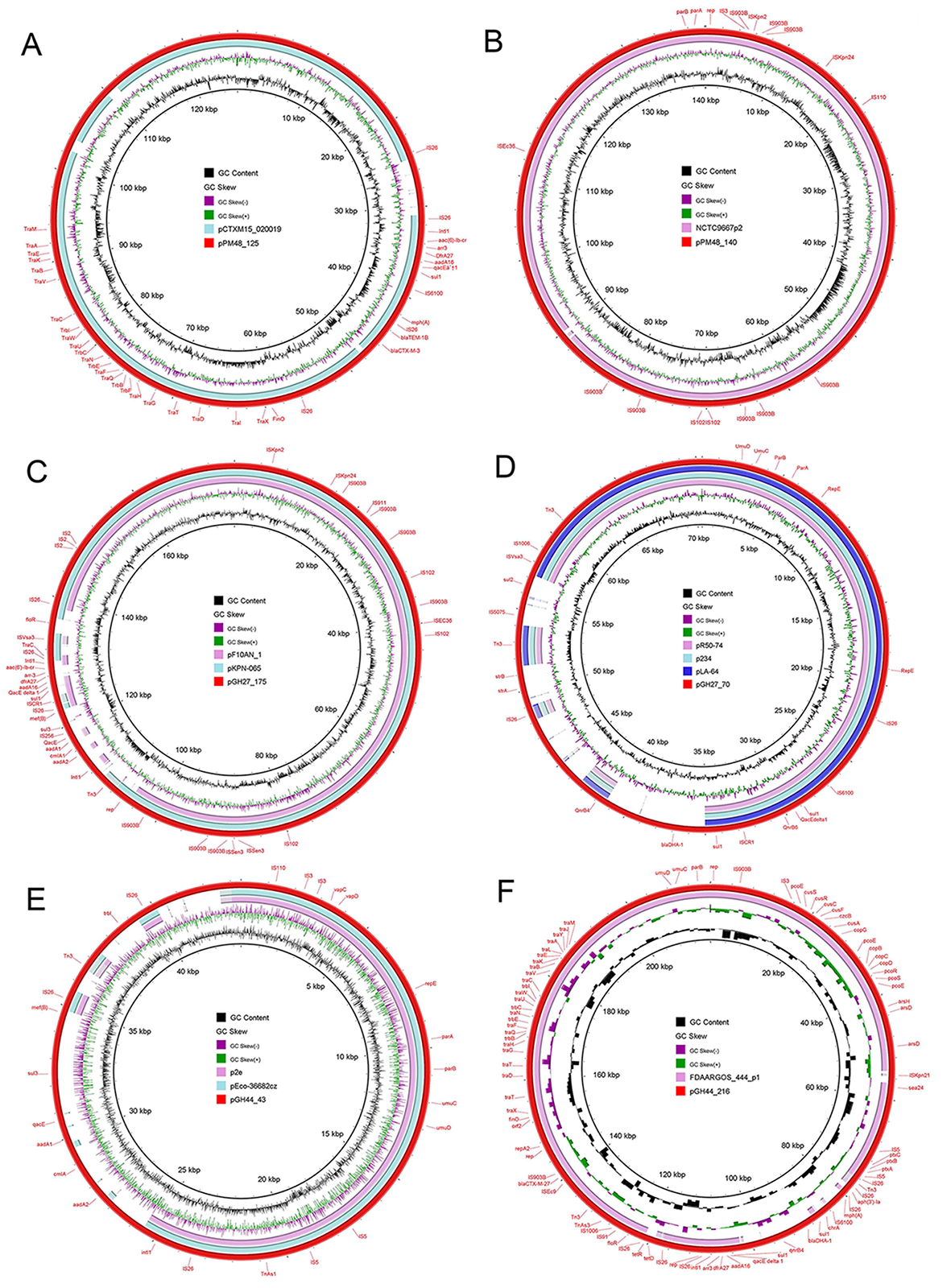

Supplement: FIG S2 [file mSystems.00140-20-sf002.tif]

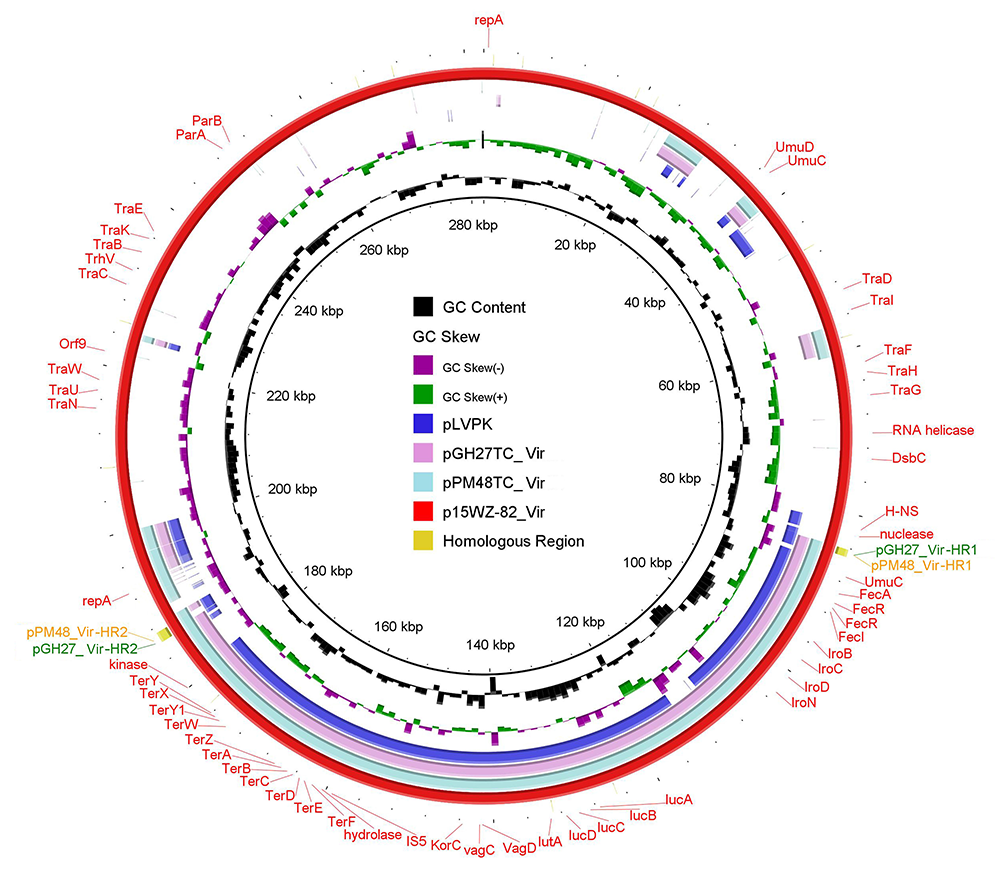

Supplement: FIG S3 [file mSystems.00140-20-sf003.tif]
